# Species traits and environmental characteristics together regulate ant‐associated biodiversity

**DOI:** 10.1002/ece3.2276

**Published:** 2016-08-18

**Authors:** Kaitlin U. Campbell, Thomas O. Crist

**Affiliations:** ^1^ Department of BiologyMiami UniversityOxfordOhio45056

**Keywords:** ants, biodiversity, mites, myrmecophile, phoresy, soil resources

## Abstract

Host‐associated organisms (e.g., parasites, commensals, and mutualists) may rely on their hosts for only a portion of their life cycle. The life‐history traits and physiology of hosts are well‐known determinants of the biodiversity of their associated organisms. The environmental context may strongly influence this interaction, but the relative roles of host traits and the environment are poorly known for host‐associated communities. We studied the roles of host traits and environmental characteristics affecting ant‐associated mites in semi‐natural constructed grasslands in agricultural landscapes of the Midwest USA. Mites are frequently found in ant nests and also riding on ants in a commensal dispersal relationship known as phoresy. During nonphoretic stages of their development, ant‐associated mites rely on soil or nest resources, which may vary depending on host traits and the environmental context of the colony. We hypothesized that mite diversity is determined by availability of suitable host ant species, soil detrital resources and texture, and habitat disturbance. Results showed that that large‐bodied and widely distributed ant species within grasslands support the most diverse mite assemblages. Mite richness and abundance were predicted by overall ant richness and grassland area, but host traits and environmental predictors varied among ant hosts: mites associated with *Aphaenogaster rudis* depended on litter depth, while *Myrmica americana* associates were predicted by host frequency and grassland age. Multivariate ordinations of mite community composition constructed with host ant species as predictors demonstrated host specialization at both the ant species and genus levels, while ordinations with environmental variables showed that ant richness, soil texture, and grassland age also contributed to mite community structure. Our results demonstrate that large‐bodied, locally abundant, and cosmopolitan ant species are especially important regulators of phoretic mite diversity and that their role as hosts is also dependent on the context of the interaction, especially soil resources, texture, site age, and area.

## Introduction

The relative importance of abiotic and biotic components in determining the distribution and abundance of organisms is well established for free‐living animals. For host‐associated organisms, such as parasites, commensals, and mutualists that rely on hosts for dispersal, food, or reproduction, the species traits of their hosts may interact with the environmental context to control their distributions and abundances. While the host individual is often treated as a conveniently delineated habitat boundary (Guégan et al. 2005; Pérez‐del‐Olmo et al. 2009), many ectoparasites, for example, move among hosts throughout their life cycle, require multiple host species, and can also be affected by biotic and abiotic constraints of their hosts’ environment (Krasnov et al. 2004). Host traits, distribution, and population density are well‐studied and significant drivers of host‐dependent organism diversity (Lindenfors et al. 2007; Poulin 2007), but the environmental context of hosts may also alter the strength and diversity of their interactions with associated organisms. For example, differences in light availability can shift an ant–plant mutualism to a commensalism (Kersch and Fonesca 2005). Similarly, wood‐boring pine beetles, dependent on microbial symbionts, can have devastating population eruptions under different climate and anthropogenic disturbances (Raffa et al. 2008), while tick‐borne Lyme disease outbreaks are structured by not only the availability of hosts, but also host food resources and climate (Ostfeld et al. 2006). Habitat loss and isolation may also be important to both hosts and their associates, as demonstrated by Ewers et al. (2013), who found that forest fragmentation and edge effects weaken a bee–mite commensalism. In essence, the environmental context of a host can alter host competency and affect the interactions and biodiversity of host‐dependent organisms.

**Figure 1:**
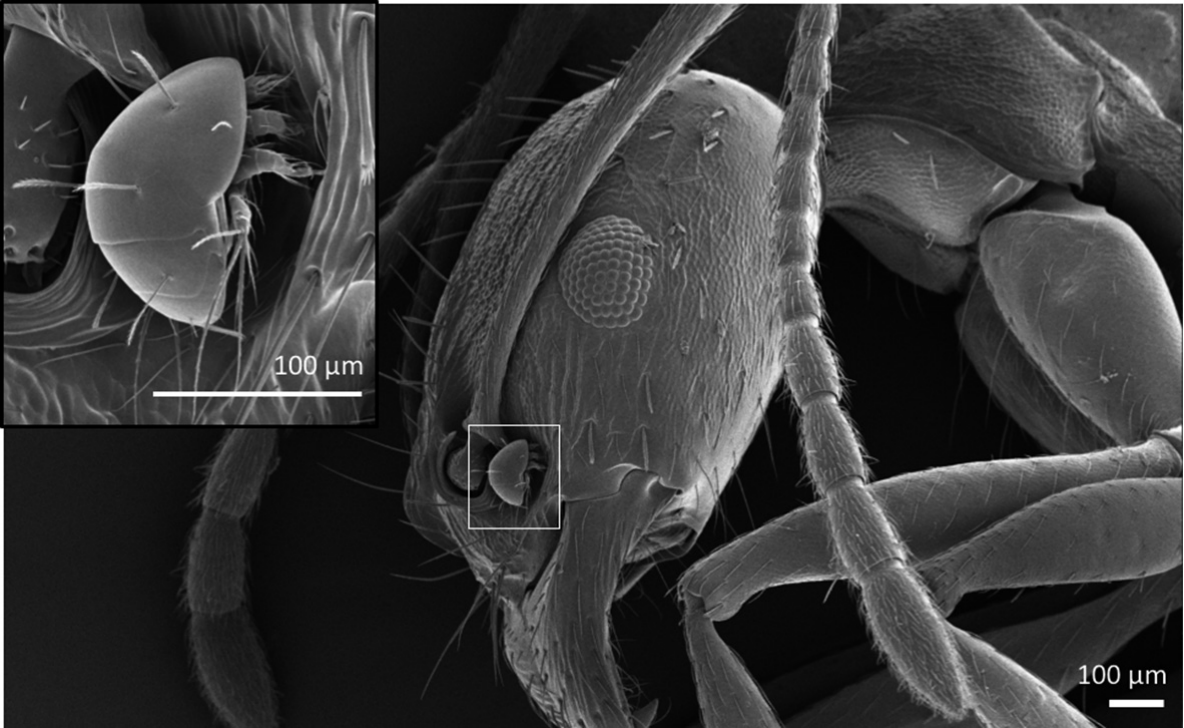
SEM micrograph of *Aphaenogaster rudis* ant host with *Scutacarus* sp. (Heterostigmata) mite phoretic in groove at the base of the antenna (enlarged in inset).

Ants host a significant diversity of associated organisms (myrmecophiles) and regulate biodiversity in above‐ and belowground food webs (Howe and Smallwood 1982; Sanders and Platner 2007; Dunham and Mikheyev 2010). Many organisms exploit resources in ant nests, for example, detritus, middens, small prey, fungi, and ant brood (Kistner 1982; Elmes et al. 1998; Rettenmeyer et al. 2011). Mites are the most frequently encountered and speciose of the myrmecophiles, but are typically overlooked, likely due to their small size and the lack of taxonomic specialists (Rettenmeyer 1962; Kistner 1979). They are frequently found riding on other animals, especially insects, in a commensal interaction, known as phoresy (Fig. 1), which facilitates mite dispersal to isolated resources (Houck and OConnor 1991).

Most ant‐associated mites rely directly on the host for phoresy during only a portion of their life cycle and use soil or ant nest resources (e.g., detritus, fungi, bacteria, and small prey) during nonphoretic life stages (Eickwort 1990). Although little is known about the ecology or function of the mites when they are not on the host, there is evidence that many ant‐associated phoretic mite species are host specific (Campbell et al. 2013) and have evolved synchronized life cycles with their hosts to optimize their dispersal opportunities (Kaliszewski et al. 1995; Moser and Blomquist 2011; Uppstrom and Klompen 2011). Host characteristics can be important drivers for myrmecophile richness. Large colonies, cosmopolitan hosts, and concentrated colonies are known to influence myrmecophilous beetle species richness (Päivinen et al. 2003, 2004). Recent studies have also identified the importance of life‐history characteristics of ant hosts (colony size, host size, and social parasitism) for phoretic mite richness and prevalence (Campbell et al. 2013).

The majority of the ant‐associated mites are not believed to be parasites based on mouthpart morphology, behavioral observations, or known ecology of closely related species, but their dependence on other species for dispersal and resources are characteristics applicable to existing ecological frameworks of parasitology. For example, large‐bodied ant species host a greater diversity of phoretic mite species (Campbell et al. 2013), a relationship that is mirrored by the parasite ecology literature involving comparisons of hosts over limited ranges of body size (Lindenfors et al. 2007; Poulin 2007). Other established principles in parasite ecology may also apply to phoretic mite communities, such as the relationship of greater parasite diversity and specialization with wide‐ranging host species in dense populations (Lindenfors et al. 2007; Harris and Dunn 2010). Parasite diversity has been suggested to indicate trophic complexity and habitat quality, because parasites are relying on the presence of other species (Hudson et al. 2006). In this way, phoretic mite diversity has the potential to indicate complexity of soil food webs or host diversity.

As with many ectoparasites, however, only a portion of the phoretic mite (or phoront) life cycle is spent on the host, so that the environmental context of the host also plays a significant role in structuring phoretic mite community composition and diversity. Few studies have tested how phoront or parasite diversity is affected by environmental characteristics or disturbance of the habitat. Ewers et al. (2013) found bumblebees occurring in larger forest fragments, higher in the canopy, and farther from the edge had higher phoretic mite loads (number of mites per host individual). A second study of fragmentation focused on burying beetles (*Nicrophorus*) and mutualistic *Poecilochirus* mites (Gibbs and Stanton 2001). *Poecilochirus* in moderate loads are beneficial because they control fly populations that compete with *Nicrophorus* larvae, but in large numbers, they begin to prey on the larvae. In fragmented forest sites, *Poecilochirus* mite loads were more often too low to be beneficial or too high, shifting to detrimental levels. A third study of parasitic gamasid mites specifically tested the relative roles of host and environment and found that parasitic mite abundance is primarily influenced by host identity and temperature, while host identity and precipitation contributed to species richness (Krasnov et al. 2008). Together these three studies indicate the importance of disturbance, habitat area, edge effects, and abiotic factors in determining the strength of the host–mite interactions and mite diversity in ways that go beyond host traits.

Here, we determined the roles of host availability and host traits in supporting biodiversity of phoretic mite communities and how they are modified by environmental characteristics of grassland patches embedded within agricultural landscapes. Specifically, we tested three hypotheses. (1) Mite diversity is determined by availability of suitable host species and soil detrital resources (e.g., organic matter, litter depth) within the grassland. This hypothesis leads to the predictions that overall phoretic mite diversity would be greatest in patches with higher abundance and richness of suitable ant hosts and at sites where preferred host ant species are more widespread. Further, we predicted that grasslands with more soil organic matter (SOM) and litter depth would have higher phoretic mite diversity. (2) Mite communities are structured by microhabitat variation in soil texture due to its effects on soil pore size, moisture, and bulk density. In addition, soil texture was predicted to influence the distribution of host ants. We predicted that mites would show patterns similar to their host ants, such that diversity is higher in sandier sites (Campbell and Crist, in review). Lastly, we hypothesized that (3) phoretic mite communities are structured by the habitat age, size, and disturbances due to their limited dispersal abilities. We predicted that smaller, more recently planted grassland sites or those with greater edge:area ratios would have lower mite abundance and richness. More recently burned sites were also predicted to have lower mite diversity because of decreases in depth of the insulating litter layer.

## Methods

### Study sites

Our study sites comprised 23 warm‐season constructed grasslands on retired agricultural fields, managed primarily by local landowners and MetroParks in Butler, Montgomery and Preble Counties of southwestern Ohio. Grasslands were planted with similar mixtures of native warm‐season grasses and forbs, but varied in size, time since planting, and management, such as fire or mowing (Table S1). In each summer of 2011 and 2012, we sampled 20 of the 23 grassland sites due to changes in voluntary landowner participation between years.

### Ant and mite collections

To assess ant species richness and distributions at each of the 23 sites, we used transects of 5–10 pitfall traps per site, with the number of traps log‐scaled to grassland area (Campbell and Crist, in review). Traps were 25 m apart and sampled three times during each summer (Figure S1). Although pitfall traps are widely used to sample ant communities, ants collected in pitfall traps cannot be reliably used for phoretic mite collections because most mites become dislodged from the ants in fluid traps, and pitfalls collect many other arthropods that may be carrying their own assemblages of phoretic mites. We therefore developed a baiting technique to collect the ants used for phoretic mite sampling. Baits tend to be unreliable for sampling the full spectrum of the ant community, because they favor more common ants with generalist diets (Albrecht and Gotelli 2001; Hahn and Wheeler 2002); however, for this study, we were less interested in the total diversity of mites associated with all ant species, but rather how the diversity of mites on more suitable and common host species changes depending on habitat characteristics, disturbance, and host frequency.

To collect the greatest diversity of ants with baits, we found it optimal to sample over multiple time intervals with both carbohydrate and protein baits (Albrecht and Gotelli 2001; Hahn and Wheeler 2002). Our preliminary studies revealed that the first ants to find the baits were typically large and highly vagile ants, which were later displaced by large numbers of small ants, essentially a dominance–discovery trade‐off. Using the same transects established for the pitfall traps, we placed 10–20 bait stations spaced 8.3 m away from each side of the pitfall traps (Figure S1). Baiting took place for an hour at each site during the same week the pitfall traps were active. The baits consisted of a small amount of fish‐flavored canned cat food and crushed pecan sandies placed on 7.6 × 12.7 cm index cards marked with three time intervals (20, 40, and 60 min). A 20‐min bait card was initially placed on bare ground at each station along the transect. After 20 min, the ant‐covered baits were placed into individual plastic zip bags and replaced with the 40‐min bait card. This process was repeated for the 60‐min card. Bait cards with ant collections were frozen until inspection for mites. A total of 930 and 924 baits were used for each sampling period in 2011 and 2012, respectively.

Bait collections were analyzed for mites by inspecting each ant for phoretic mites under a dissecting microscope. All mites associated with the ants were placed in lactic acid to clear internal structures and mounted on slides in Hoyer's or polyvinyl alcohol media. The majority of ant‐associated mites are undescribed; therefore, mites were identified to genus and morphospecies, and whenever possible, to species. Voucher specimens of each mite and ant species are deposited in the Ohio State Acarology and Triplehorn Insect Collections, respectively.

### Soil sampling and processing

Our first two hypotheses involve the role of soil characteristics as measures of detrital food resources and microhabitat suitability. We collected soil cores (10 cm deep, 5 cm diameter) to assess multiple soil characteristics (SOM, soil texture, and bulk density) to test our first two hypotheses. To measure soil detrital food resources, we used the average depth of the litter layer near each bait station and the percent SOM. SOM was determined in the laboratory. Soil samples were crumbled, sieved, finely ground with a mortar and pestle to reduce small clay aggregates, and homogenized for subsampling. We used 20‐g subsamples for organic matter quantification. SOM samples were oven‐dried, weighed, burned in muffle furnace (450°C for 8 h), and then reweighed. Percent organic matter is the percent weight lost due to burning.

Soil bulk density and texture were hypothesized to influence the microhabitat suitability for mites and their hosts. Soil bulk density was determined by air‐drying each soil core and taking the final weight and volume of the core. After processing the air‐dried soil cores into homogenized samples, 40 g of soil was subsampled for soil texture analysis. Soil texture (percent sand, silt, and clay) was measured using the hydrometer method approach (Sheldrick and Wang 1993). This method relies on separation of sand, silt, and clay particles from the solution due to differences in particle mass.

### Analysis of host suitability and soil detrital resources

Our first hypothesis aimed to identify the roles of host suitability and soil detrital resources on mite diversity. We compared host suitability of ant species using generalized linear models (GLM) with ant host size (worker length) and abundance (number of ant workers inspected) as predictors for associated mite richness. We conducted this analysis using ants from all sites pooled together and only included ant species for which at least 30 individuals had been inspected. We removed *Monomorium minimum* from the analysis due to unusually large sample sizes (>80,000 individuals) and very low mite prevalence (<0.02%). Previous studies have shown that larger hosts support greater mite diversity and number of hosts inspected can be an important measure of sampling effort (Campbell et al. 2013). Average worker size was extracted from Coovert (2005).

To test whether shifts in ant and mite community composition were correlated among sites, we used a Mantel test, which compares dissimilarity between two matrices (mantel function, vegan package of R) (Oksanen et al. 2013). If there is a correlation in community dissimilarity, this can mean either that these two communities are responding to each other or that they covary in species composition due to a common environmental factor(s). The Mantel test does not partition the variance in the communities. Therefore, we conducted an unconstrained ordination to test the role of host identity (as a site score). We conducted a multidimensional scaling (MDS) analysis (mds function, vegan package, R) with Bray–Curtis dissimilarity (vegdist function, vegan package, R) (Oksanen et al. 2013) on the mite communities (41 mite species total) associated with the seven common ant species that carried the majority of the mites (97.5% of mite abundance). Ant species were only included if they carried mites at a minimum of four sites. We used a permutational ANOVA to test host species identity as a predictor of mite community composition with site as stratum (adonis function, vegan package) (Oksanen et al. 2013).

### Disturbance and habitat characteristics

Our third hypothesis tested the effects of disturbance and other habitat characteristics on phoretic mite diversity. We used the following predictors as measures of disturbance: age (time since planting) and time since burn (management), and additional measures of habitat included ratio of edge to area and area. We tested these factors with (see also Combined Effects section below) and without the effects of host species. To control for variation in host species traits, we tested the predictors for mite assemblages associated with two common host species, *Aphaenogaster rudis* (Fig. 1) and *Myrmica americana*. For *A. rudis* and *M. americana* mite analyses, we combined the 2 years of data due to low sample sizes.

### Combined effects of host and habitat

Both host and habitat characteristics likely play important roles in structuring the mite communities; therefore, we combined host and environmental predictors when constructing our GLMs for overall mite richness and mite abundance at the site level. Potential variables for models included ant species richness (from pitfall traps), soil texture, SOM, bulk density, litter depth, age, time since burn, area, and edge:area. We log‐transformed age, time since burn, and area predictors.

We also conducted separate analyses for richness and abundance of two major mite taxonomic groups: cohorts Astigmata and Heterostigmata. These two taxa differ in their phoretic stage, host specificity, and sensitivity to habitat and resources (Campbell et al. 2013). Astigmata associated with these ant species are phoretic as deutonymphs (an immature stage of development) and eat a wide range of resources in nonphoretic stages (Houck and OConnor 1991). Heterostigmata associated with these ant species enter phoresy as specialized adult females (Fig. 1) and eat fungi when nonphoretic (Binns 1982). Previous studies found that Heterostigmata are more host specific with 61% associated with a single ant species and 35% on hosts in the same genus; in contrast, 40% of Astigmata species are associated with a single ant species and 44% were associated with multiple congeneric ant hosts (Campbell et al. 2013). Due to differences in host specificity, we expected Astigmata abundance and richness to be primarily determined by environmental characteristics (disturbance or soil resources) rather than ant species richness within a site. In contrast, we expected Heterostigmata richness would be determined by ant species richness and host frequency within sites, while mite abundance would more influenced by disturbance or soil resources.

To determine the role of host richness and habitat characteristics on mite community composition among sites, we conducted a constrained ordination using specific environmental variables in a distance‐based redundancy analysis (dbRDA) with Bray–Curtis dissimilarity (McArdle and Anderson 2001). We used Akaike's information criterion (AIC) to select the best‐fitting ordination model and obtained *P*‐values using random permutations (999 permutations). The dbRDA was conducted with a user‐written function in R (M. Anderson, pers. comm.).

We used GLMs in the glm function of the R programming language (R Development Core Team 2015) with Poisson error distributions for all richness models and Gaussian error distributions for log‐transformed abundance. GLMs were conducted separately for the two study years (2011 and 2012) due to three sites that differed between the years except for the tests of mites associated with *M. americana* and *A. rudis* and our composition analyses (dbRDA and MDS) in which we pooled the 2 years of data. As an alternative approach, we tested for indirect effects of habitat characteristics mediated through ant hosts and direct effects on mite diversity using piecewise structural equation models (SEM). We compared our GLM with the SEM approach for 16 models: abundance and richness for all mite taxa in 2011 and 2012 (four models), for the Astigmata and Heterostigmata mite taxa in 2011 and 2012 (eight models), and for mites associated with two focal ant species (four models). We selected the best‐fitting SEM models using AICc and tested for missing paths using Fisher's C. This approach provided no additional insights and when compared with GLM's were of a poorer fit. We thus focus from here on the GLMs.

The numbers of baits and pitfall traps were scaled to the log area of each site to account for heterogeneity in larger sites; we therefore verified that relationships with habitat area were not due simply to differences in sampling intensity. We conducted a subsampling of baits from each site in which only 10 baits or five pitfall traps (the number used in the smallest sites) were randomly selected 1000 times. The species abundance values for each randomization were averaged and the richness was calculated at each site. Mite species richness of all subsampled sites was unchanged compared to that of the larger data, except for a single site that lost a rare species. Ant richness was also unchanged from the larger data; therefore, we continued to use the full data for mite and ant richness in subsequent analyses.

The best‐fitting linear models were selected using minimum AIC. Any models differing by ≤2 AIC points were considered competing models. When comparing competing models, best models were those with the lowest AIC scores and the least number of predictors. If a competing model had a lower AIC score with an additional predictor, we conducted a likelihood ratio test versus the reduced model to determine whether the additional predictor significantly improved the model fit. In all cases, additional predictors in competing models did not significantly improve the models (*P* < 0.05). We tested best‐fitting models against the null model (intercept only) in a likelihood ratio test to calculate *P*‐values and to determine the percent deviance explained as a measure of model fit.

## Results

A total of 104,766 ants belonging to 27 ant species were collected with baits and individually inspected for phoretic mites (Table S2). *Monomorium minimum*, a small competitive ant species that overwhelmed the baits during the latter part of the 60‐min trials, comprised 82,521 (78%) of the ants and mite prevalence was only 0.02% (16 ants with mites). A total of 51 species of mites (1584 individuals) were collected from 15 ant host species (1047 ant individuals) (Tables S2 and S3). Mite richness included 20 Astigmata species (1201 individuals), 27 Heterostigmata species (377 individuals), and four Mesostigmata species (six individuals). Average mite richness and abundance per site were 7.6 species (range = 0–16) and 68.9 individuals (range = 0–208), respectively.

Pitfall ant collections, used for ant species richness in our analysis, comprised 31 ant species including eight species not collected by baiting. These eight additional ant species were uncommon or rare species, and because they did not approach the baits we were unable to assess their associated mite diversity.

### Role of host suitability and identity

**Figure 2:**
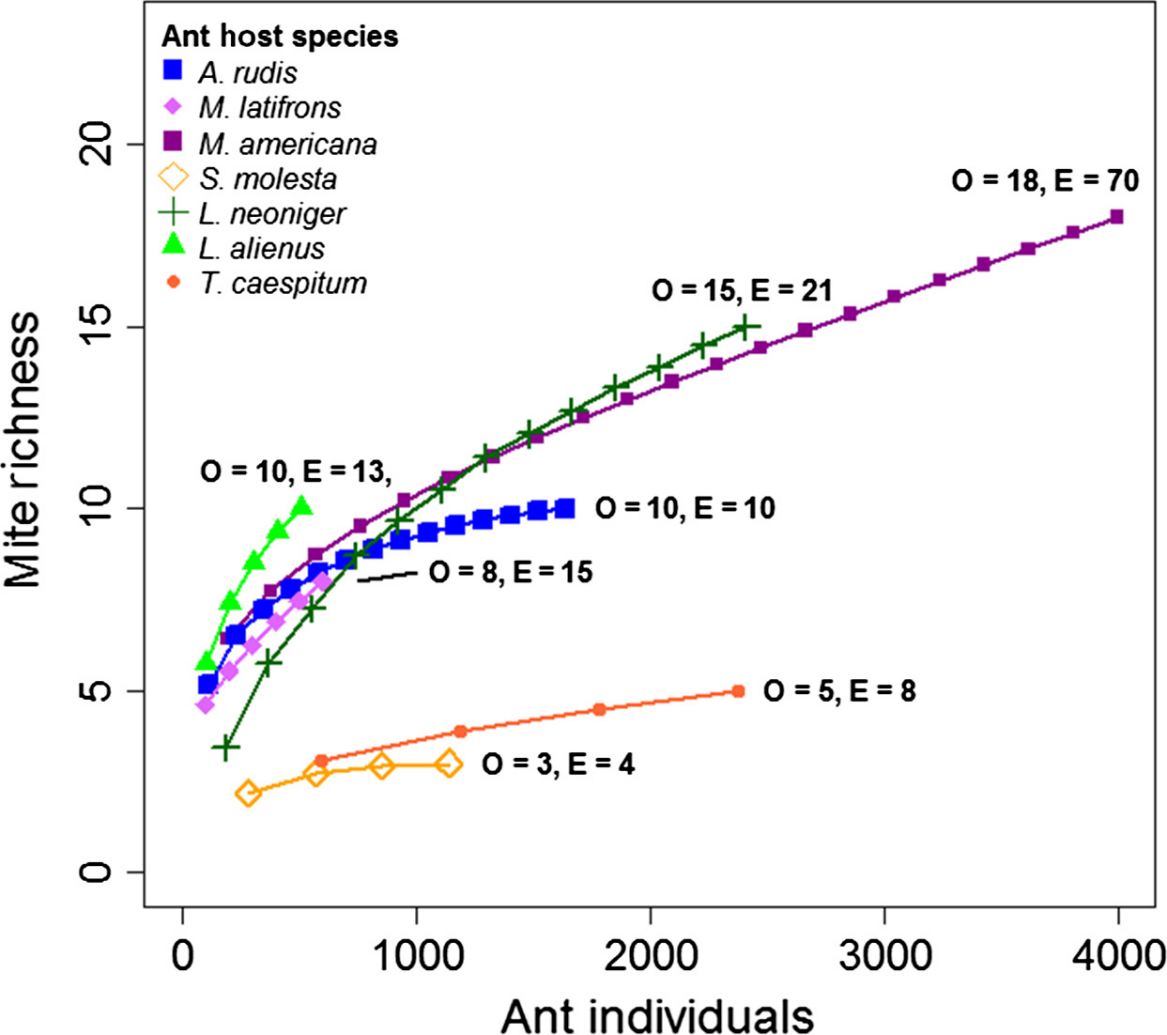
Species accumulation curve of mite species by number of host ant individuals inspected. Number of points on a curve represents the number of sites where the ant host was collected, while length of the curve represents the number of ant individuals inspected. Ant species that are more cosmopolitan (at more sites) and more abundant also have higher observed (O) and Chao1 estimated (E) species richness.

There was an average of 6.6 mite species associated with each ant species (excluding *M. minimum*); however, some ant species hosted much greater diversity, such as *Myrmica americana,* with 18 species and 937 mite individuals (Table S1). We used a species accumulation curve to summarize the observed and Chao 1 estimate (Chao et al. 2005) of mite species for seven common ant hosts that carried 97.5% of the overall mite abundance and 77.4% of the total richness (Fig. 2). Ant species that were more cosmopolitan (at more sites) and more abundant tended to have higher observed and estimated species richness. The best model for predicting mite richness among host species (host suitability) included host body size and host abundance (*P* < 0.0001, Dev. expl. = 63.0%).

**Figure 3:**
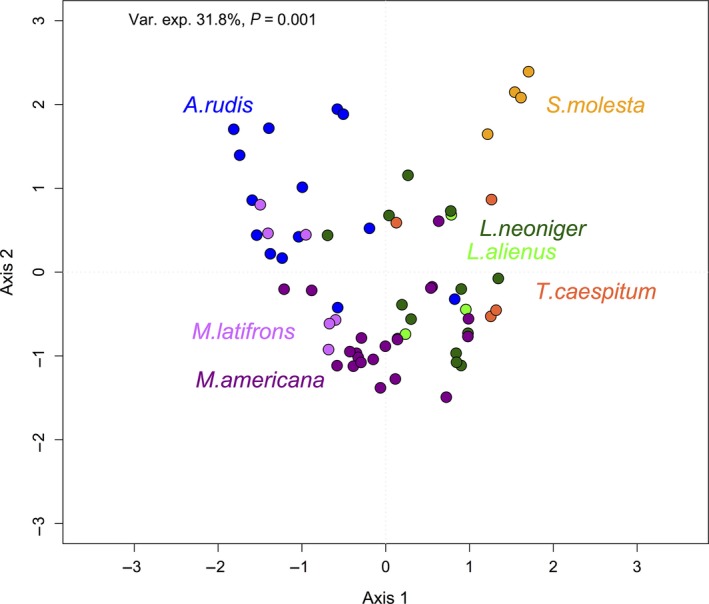
Multidimensional scaling ordination of mite community composition. Host identity was a significant predictor of mite community (*P* = 0.001, Variance expl. = 31.8%). Mite communities separate by host species and cluster by host genus (e.g., *Myrmica americana* and *Myrmica latifrons*,* Lasius neoniger,* and *Lasius alienus*).

There was a significant positive relationship between the ant and mite community dissimilarity (*P* = 0.001, Mantel *r* = 0.51), suggesting that ant hosts and associated mites have similar patterns of turnover in species composition among sites. Host ant species explained a significant amount of the variance in mite species dissimilarity, as shown by MDS ordinations of mite communities for seven host species (*P* = 0.001, Var. expl. = 31.8%). Host specificity by mites at both species and genus levels was indicated by clustering in multivariate space (Fig. 3).

### Controlling for host identity – role of soil resources and disturbance

To understand how resources and disturbance affect mite assemblages while holding host traits constant, we used a subset of the mite community associated with *M. americana* and *A. rudis*. Richness and abundance of mites associated with *A. rudis* were best explained by litter depth (*P* = 0.0003, Dev. expl. = 31.6% and *P* = 0.003, Dev. expl. = 33.2%, df = 1,17, respectively) (Table 1). Competing models included host frequency and ant richness (Table 1). Mite richness and abundance on *M. americana* were best explained by ant host frequency within sites and site age (*P* = 0.0003, Dev. expl. = 31.6% and *P* = 0.003, Dev. expl. = 33.2%, df = 2,20, respectively) and there were no competing models (Table 1).

**Table 1:** Best and competing models (ΔAIC < 2) for richness and abundance for mites associated with *Aphaenogaster rudis* and *Myrmica americana*

Response	Best model	ΔAIC vs. null	% Dev. Expl.	Competing models	ΔAIC vs. best	% Dev. Expl.
Richness						
* rudis*	(+)Litter depth	−10.90	31.6	(+)Litter depth + (+)Host Freq.	−1.32	39.8
* americana*	(+)Host Freq. + (+)Log (age)	−25.61	55.4	–	–	–
Abundance						
* rudis*	(+)Litter depth	−5.64	33.2	(+)Litter depth + (+)Ant richness	−0.81	42.4
* americana*	(+)Host Freq. + (+)Log (age)	−18.44	62.3	–	–	–

AIC, Akaike's information criterion.

### Combined effects of host and environmental characteristics

The best‐fitting model for 2011 mite species richness was ant richness (*P* = 0.001, Dev. expl. = 19.3%, df = 1,18) (Table 2). A competing model included area in addition to ant richness (ΔAIC = −1.39, Dev. expl. = 25.5%, df = 2,17). The best model for 2012 was area (*P* = 0.019, Dev. expl. = 18.8%, df = 1,18) (Table 2) and a competing model included ant richness (ΔAIC = −0.11, Dev. expl. = 21.4%, df = 2,17). Overall mite abundance for 2011 was best predicted by ant richness (*P* = 0.0003, Dev. expl. = 41.8%, df = 1,18) (Table 2) and a competing model also included age (ΔAIC = +1.15, Dev. expl. = 41.8%, df = 2,17). In 2012, there were no models that were better than the null model. Thus, overall mite species richness and abundance were best explained by either host species richness or habitat area.

**Table 2:** Best and competing models (ΔAIC < 2) for overall, Astigmata, and Heterostigmata richness and abundance

Response	Best model	ΔAIC vs. null	% Dev. Expl.	Competing models	ΔAIC vs. best	% Dev. Expl.
Overall mite richness						
2011	(+)Ant richness	−8.72	19.3	(+)Ant richness + (+)Log (area)	−1.39	25.5
2012	(+)Log (area)	−3.46	18.8	(+)Ant richness + (+)Log (area)	+0.11	21.4
Overall mite abundance						
2011	(+)Ant richness	−8.823	41.8	(+)Ant richness + (+)Log(age)	+1.15	41.8
2012	Null	–	–	–	–	–
Astigmata richness						
2011	(+)Log (area)	−3.68	22.0	(+)Log (area) + (+)Ant richness	−0.10	30.1
				(+)Ant richness	+0.79	18.9
				(+)Log (age)	+1.60	15.8
2012	Null	–	–	(+)Litter depth	+0.11	5.2
Astigmata abundance						
2011	(+)Ant richness	−5.42	31.0	(+)Ant richness + (+)Log (age)	−0.25	38.3
				(+)Log (age)	+1.24	26.6
2012	Null	–	–	(+)Log (area)	−0.03	9.6
Heterostigmata richness						
2011	(+)Ant richness	−2.81	11.8	(+)Ant richness + (+)Log (area)	−0.05	16.8
				(+)Log (area)	+0.32	11.0
2012	(+)Log (area)	−3.42	24.4	(+)Log (area) + (+)Ant richness	+1.56	24.5
Heterostigmata abundance						
2011	(+)Ant richness	−6.52	34.7	(+)Ant richness + (+)Log(age)	+1.73	35.6
2012	Null	–	–	–	–	–

AIC, Akaike's information criterion.

**Figure 4:**
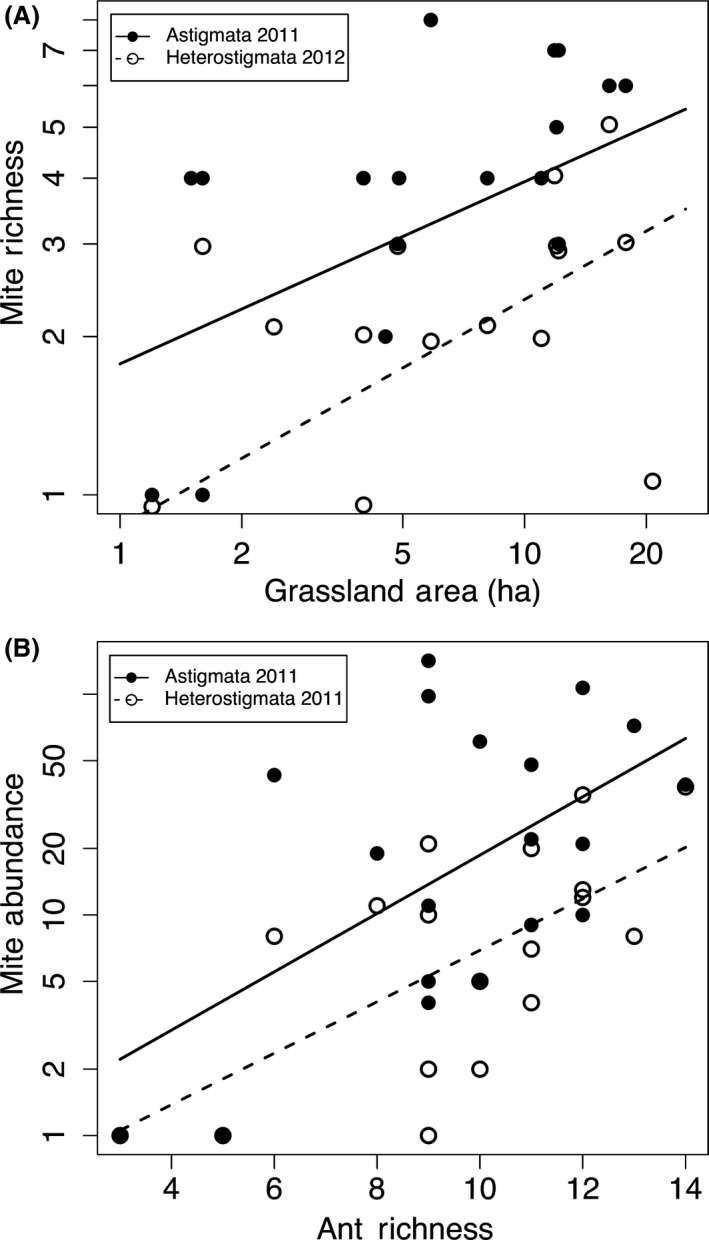
Best‐fitting models for (A) 2011 Astigmata (solid line, filled circles) and 2012 Heterostigmata (dashed line, open circles) richness included grassland area (ha), and (B) abundance of both taxa (2011) was best predicted by ant richness. Note: axes presented on log scale.

Astigmata richness in 2011 was best predicted by area (*P* = 0.017, Dev. expl. = 22.0%, df = 1,18) (Fig. 4A) and there were multiple competing models including ant richness as well as age (Table 2). The 2012 best model was the null model and the only competing model was litter depth, which explained very little and was not significant (*P* = 0.169, Dev. expl. = 5.2%, df = 1,18). Heterostigmata richness in 2011 was best predicted by ant richness (*P* = 0.028, Dev. expl. = 11.8%, df = 1,18), with multiple competing models, and in 2012, area was the best model (*P* = 0.02, Dev. expl. = 24.4%, df = 1,18) (Fig. 4A, Table 2) with area and ant richness as a competing model. Abundance for both Astigmata (*P* = 0.004, Dev. expl. = 31.0%, df = 1,18) and Heterostigmata (*P* = 0.002, Dev. expl. = 34.7%, df = 1,18) was best predicted by ant richness in 2011 (Fig. 4B) and competing models for both also included age (Table 2). In 2012, there were no models better than the null for abundance of either mite taxon. As with overall mite richness and abundance, the two most important predictors for these two taxa were species richness of ant hosts and habitat area.

**Figure 5:**
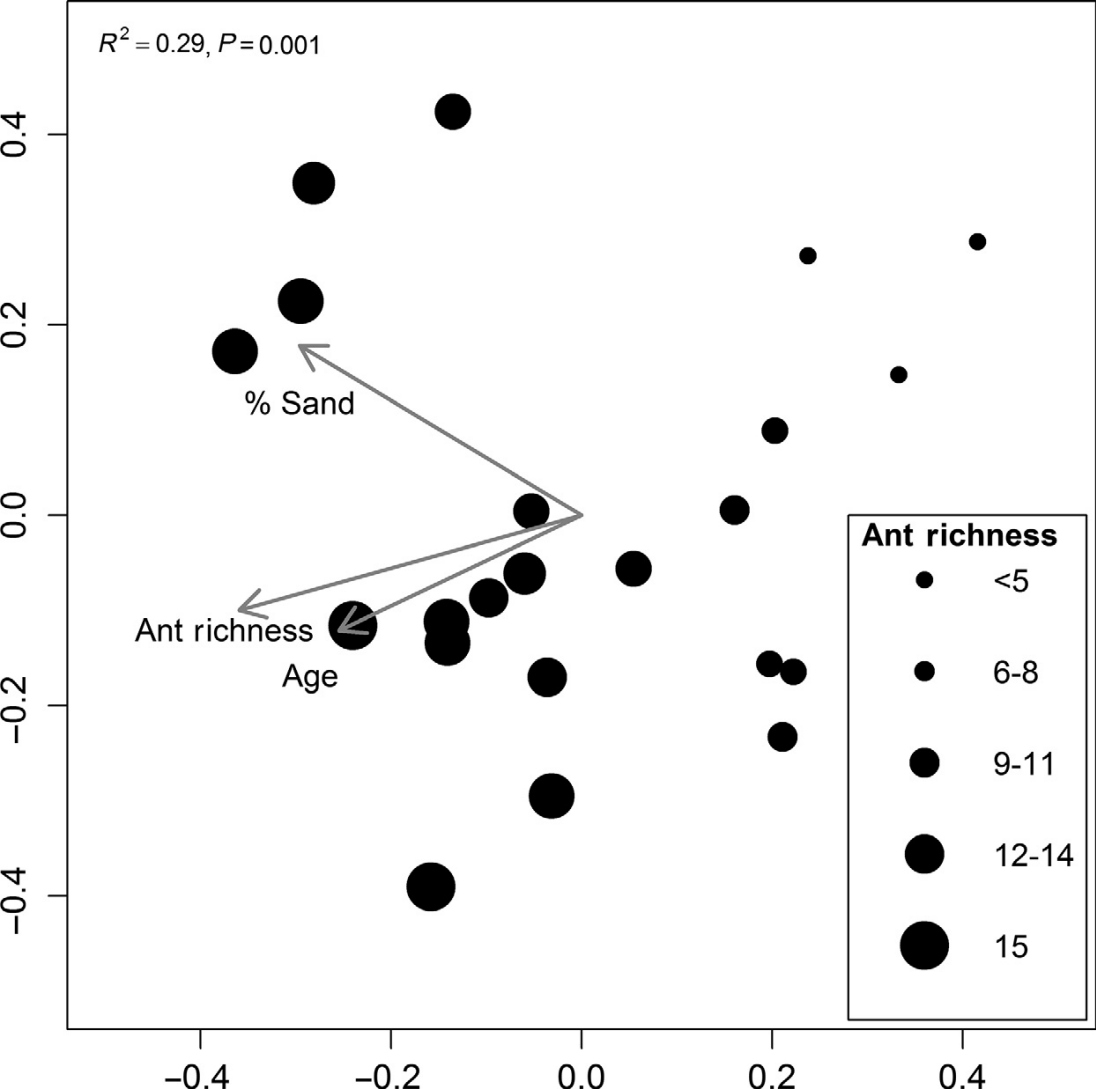
Akaike's information criterion selected distance‐based redundancy analysis multivariate ordination of mite community composition across 23 grasslands. Symbols indicate site scores of grasslands and are sized according to the most important predictor variable (ant richness). Arrows are biplot correlations of the significant predictor variables (ant richness, % sand, and site age).

Constrained ordinations using host and environmental variables showed that the best model for mite community composition using dbRDA included ant richness, age, and percent sand (Pseudo *F* = 2.50, *P* = 0.001, *R* ^2^ = 0.29) (Fig. 5).

## Discussion

The objective of this study was to determine how the environmental context of ant hosts may affect their role as regulators of mite diversity. Using GLMs and community composition analyses, we found that both host identity and environmental characteristics play significant roles in structuring ant‐associated mite diversity and community composition in grasslands.

### Host suitability, frequency, and richness

Ant‐associated mite diversity was dependent on host suitability and frequency of occurrence in constructed grasslands. We documented 51 phoretic mites on 15 ant species in the grasslands; however, 12 ant species did not carry any mite species. It is clear that some ant species in the grassland are more suitable hosts than others, but what makes a given ant species a more preferred host? It is already established in this phoretic mite system and multiple parasite systems that larger‐bodied hosts have higher associated species richness (Lindenfors et al. 2007; Poulin 2007; Campbell et al. 2013). We provided additional evidence of this relationship with host size and also found that host frequency within and host frequency among sites are also important drivers of phoretic mite diversity. The latter findings support established principles in parasite ecology that greater parasite diversity occurs on wide‐ranging host species with dense populations (Lindenfors et al. 2007; Harris and Dunn 2010).

Ant richness was the best predictor for overall mite richness and abundance, Astigmata abundance, and Heterostigmata richness and abundance (Fig. 4B). Interestingly, mites were not found on the less common ant species that contribute to higher ant species richness at a given site; instead, common ants seem to be accumulating more mite species in sites with higher ant richness. This suggests that both mite and ant richness may be exhibiting covarying responses to environmental factors, for example, grassland age and soil texture (Campbell and Crist, in review). Further, the Mantel test of ant and mite community dissimilarities showed a strong relationship in turnover of these two communities, and the dbRDA provided support for the importance of ant richness, soil texture, and age in explaining the variation in composition of the mite communities (Fig. 5).

### Disturbance, soil, and habitat characteristics

We expected ant‐associated mite biodiversity to be filtered, in part, by host‐mediated colonization of the grasslands and to show similarities to free‐living soil mite communities that are largely driven by patterns of disturbance and soil resource availability. The most important habitat characteristics affecting overall mite biodiversity were area, age, litter depth, and soil texture. As in other phoretic mite studies, smaller habitat area decreased diversity (Ewers et al. 2013). Species–area relationships are well established in ecological theory across an array of animal species (Watling and Donnelly 2006). Species–area relationships with mites, although very limited in number and usually at a small spatial scale, are also documented for free‐living taxa (Giller 1996; Lindo and Winchester 2007). For mites associated with a single host, *M. americana*, richness and abundance of mites increased among grasslands of different ages. Similarly, mite community composition was also partly predicted by grassland age. Belowground resources and soil characteristics change as constructed grasslands mature: bulk density decreases and aggregate stability, carbon, nitrogen, SOM, microbial biomass, and fungal hyphae increase (Karlen et al. 1999; Baer et al. 2000, 2002; McLauchlan et al. 2006). Our findings provide support for successional turnover in grassland mite communities as was previously found in grassland ants (Campbell and Crist, in review, Dauber and Wolters 2005; Phipps 2006) and Collembola communities (Brand and Dunn 1998).

Time since burn, another disturbance measure did not appear in any of the best models and seems to play little role in ant‐associated mite diversity. Previous studies have demonstrated that in the short term, periodic burning decreases litter and soil moisture while increasing soil temperatures, root production, and microbial activity (Seastedt 1984). The boost in resources from fire can temporarily increase microarthropod density (Lussenhop 1976); however, the belowground resources are gradually depleted and microarthropod density decreases until the litter layer accumulates (Seastedt 1984). The complexity of responses and variation in time for this management practice could be the reason why we were unable to detect a clear signal of time since burn in our study. Our previous work found that *M. americana* frequency decreases with more recent burns (Campbell and Crist, in review). Time since burn could indirectly affect mites associated with this important host species because they respond to the host frequency of occurrence.

Soil texture was an important predictor for mite community composition among sites, but did not appear in any of the best models for abundance and richness. This may reflect species‐specific filtering of the mite community due to different soil moisture or pore size requirements. Soil samples were purposely not taken from ant nests, because ant colonies are typically long lived, and over time nests become highly altered environments with lower bulk density, higher SOM, and differing structures from the surrounding soils. It is possible that ant modifications to the nest soil could be more important to mites than many of surrounding soil characteristics.

The role of soil resource quantity (i.e., litter depth, SOM) was not apparent for overall mite richness and abundance; however, mites associated with *A. rudis* showed increased richness and abundance in sites that had greater litter depth. Mites associated with *M. americana* were not affected by litter depth and instead were driven by host frequency and age of the habitat. One explanation for this may be that the majority of the mites associated with *A. rudis* were Heterostigmata (70%) while mites associated with *M. americana* were primarily Astigmata (94%). We predicted Astigmata abundance and richness would be related to soil detrital resources because they are less host specific, while Heterostigmata richness would be determined by ant richness or host frequency and abundance by soil resources. Our results for mites on these two ant species did not support our predictions for richness and, in fact, showed the reverse: Richness in Astigmata‐dominated communities (*M. americana* mites) is primarily determined by host frequency and time since planting (age), while richness in Heterostigmata‐dominated communities is driven by litter depth. Across all ant species, Astigmata richness (2011) and Heterostigmata richness (2012) were determined by grassland area (Fig. 4), while abundance of both taxa and Heterostigmata richness in 2011 were predicted by ant richness. The strong relationship with ant richness is likely due to clear patterns of host specificity (68% of the mites in our study believed to be host specific) and importance of host identity for phoretic mite community composition that override soil resource dependence. It remains unclear as to why particular host species have such different mite communities associated with them, but this may be due to coevolutionary constraints or differences in microclimate, fungi and bacterial resources, or other ant‐dependent organisms within the nests.

Taken together, these results show that the species richness and composition of ant hosts, habitat area, and soil characteristics are all important determinants of ant‐associated soil mite communities. These predictors, however, did not explain more than 45% of the variation on mite abundance, species richness, or composition, suggesting that stochastic processes, unmeasured environmental variables, and small scale heterogeneity are also important (Caruso et al. 2012). Mites are thought to be strongly limited by dispersal (Lindo et al. 2008; Lindo and Winchester 2009), and low population sizes of different mite species may lead to frequent local extinctions within ant nests.

## Conclusions

Our study demonstrates that large‐bodied, locally abundant, and cosmopolitan ant species are especially important regulators of phoretic mite diversity. We also found that the environmental context of the host, especially soil litter, larger and older sites, can influence mite diversity and community composition. Mite and ant community composition are both structured by site age and soil texture, and ant richness is often predictive of mite richness providing support for ants as mite biodiversity indicators in constructed grasslands. Ant‐associated mite communities represent a potential model system for understanding both coevolution and assembly processes that occur in spatially isolated patches.

## Conflict of Interest

None declared.

## Supporting information


**Figure S1.** Diagram of sampling methods. Pitfall traps and vegetation quadrats were spaced 25 m apart along the transect.
**Table S1.** Descriptive table of patch‐level variables. Divisions represent general site characteristics, disturbance, and soil variables.
**Table S2.** Abundance and richness of associated mites for ant species collected with baits.
**Table S3.** Mite species collected at the 23 grassland sites.

## References

[ece32276-bib-0001] Albrecht, M. , and N. J. Gotelli . 2001 Spatial and temporal niche partitioning in grassland ants. Oecologia 126:134–141. 10.1007/s004420000494 28547432

[ece32276-bib-0002] Baer, S. G. , C. W. Rice , and J. M. Blair . 2000 Assessment of soil quality in fields with short and long term enrollment in the CRP. J. Soil Water Conserv. 55:142–146.

[ece32276-bib-0003] Baer, S. G. , D. J. Kitchen , J. M. Blair , and C. W. Rice . 2002 Changes in ecosystem structure and function along a chronosequence of restored grasslands. Ecol. Appl. 12:1688–1701.

[ece32276-bib-0004] Binns, E. S. 1982 Phoresy as migration ‐ some functional aspects of phoresy in mites. Biol. Rev. 57:571–620.

[ece32276-bib-0005] Brand, R. H. , and C. P. Dunn . 1998 Diversity and abundance of springtails (Insecta: Collembola) in native and restored tallgrass prairies. Am. Midl. Nat. 139:235–242.

[ece32276-bib-0500] Campbell, K. U. , and T. O. Crist . In review. Ant species assembly in constructed grasslands is structured at patch and landscape levels. Insect Conserv. Divers.

[ece32276-bib-0006] Campbell, K. U. , H. Klompen , and T. O. Crist . 2013 The diversity and host specificity of mites associated with ants: the roles of ecological and life‐history traits of ant hosts. Insectes Soc. 60:31–41.

[ece32276-bib-0007] Caruso, T. , M. Taormina , and M. Migliorini . 2012 Relative role of deterministic and stochastic determinants of soil animal community: a spatially explicit analysis of oribatid mites. J. Anim. Ecol. 81:214–221. 21722106 10.1111/j.1365-2656.2011.01886.x

[ece32276-bib-0008] Chao, A. , R. L. Chazdon , R. K. Colwell , and T. Shen . 2005 A new statistical approach for assessing similarity of species composition with incidence and abundance data. Ecol. Lett. 8:148–159.

[ece32276-bib-0009] Coovert, G. A. 2005 The ants of Ohio. Ohio Biological Survey, Inc., Columbus, OH.

[ece32276-bib-0010] Dauber, J. , and V. Wolters . 2005 Colonization of temperate grassland by ants. Basic Appl. Ecol. 6:83–91.

[ece32276-bib-0011] Dunham, A. E. , and A. S. Mikheyev . 2010 Influence of an invasive ant on grazing and detrital communities and nutrient fluxes in a tropical forest. Divers. Distrib. 16:33–42.

[ece32276-bib-0012] Eickwort, G. C. 1990 Associations of mites with social insects. Annu. Rev. Entomol. 35:469–488.

[ece32276-bib-0013] Elmes, G. W. , J. A. Thomas , J. C. Wardlaw , M. E. Hochberg , R. T. Clarke , and D. J. Simcox . 1998 The ecology of *Myrmica* ants in relations to the conservation of *Maculinea* butterflies. J. Insect Conserv. 2:67–78.

[ece32276-bib-0014] Ewers, R. M. , S. Bartlam , and R. K. Didham . 2013 Altered species interactions at forest edges: contrasting edge effects on bumble bees and their phoretic mite loads in temperate forest remnants. Insect Conserv. Divers. 6:598–606.

[ece32276-bib-0015] Gibbs, J. P. , and E. J. Stanton . 2001 Habitat fragmentation and arthropod community change: carrion beetles, phoretic mites, and flies. Ecol. Appl. 11:79–85.

[ece32276-bib-0016] Giller, P. S. 1996 The diversity of soil communities, the “poor man's tropical rainforest”. Biodivers. Conserv. 168:135–168.

[ece32276-bib-0017] Guégan, J. F. , S. Morand , and R. Poulin . 2005 Are there general laws in parasite community ecology? The emergence of spatial parasitology and epidemiology Pp. 22–42 *in* Renaud F. T. F., Guégan J. F., eds. Parasitism and ecosystems. Oxford Univ. Press, Oxford, UK.

[ece32276-bib-0018] Hahn, D. A. , and D. E. Wheeler . 2002 Seasonal foraging activity and bait preferences of ants on Barro Colorado Island, Panama. Biotropica 34:348–356.

[ece32276-bib-0019] Harris, N. C. , and R. R. Dunn . 2010 Using host associations to predict spatial patterns in the species richness of the parasites of North American carnivores. Ecol. Lett. 13:1411–1418. 20875037 10.1111/j.1461-0248.2010.01527.x

[ece32276-bib-0020] Houck, M. A. , and B. M. OConnor . 1991 Ecological and evolutionary significance of phoresy in the Astigmata. Annu. Rev. Entomol. 36:611–636.

[ece32276-bib-0021] Howe, H. E. , and J. Smallwood . 1982 Ecology of seed dispersal. Annu. Rev. Ecol. Syst. 13:201–228.

[ece32276-bib-0022] Hudson, P. J. , A. P. Dobson , and K. D. Lafferty . 2006 Is a healthy ecosystem one that is rich in parasites? Trends Ecol. Evol. 21:381–385. 16713014 10.1016/j.tree.2006.04.007

[ece32276-bib-0023] Kaliszewski, M. , F. Athias‐Binche , and E. E. Lindquist . 1995 Parasitism and parasitoidism in Tarsonemina (Acari: Heterostigmata) and evolutionary considerations. Adv. Parasitol. 35:335–367. 7709855 10.1016/s0065-308x(08)60074-3

[ece32276-bib-0024] Karlen, D. L. , M. J. Rosek , J. C. Gardner , D. L. Allan , M. J. Alms , D. F. Bezdicek , et al. 1999 Conservation Reserve Program effects on soil quality indicators. J. Soil Water Conserv. 54:439–444.

[ece32276-bib-0025] Kersch, M. F. , and C. Fonesca . 2005 Abiotic factors and the conditional outcome of an ant‐plant mutualism. Ecology 86:2117–2126.

[ece32276-bib-0026] Kistner, D. H. 1979 Social and evolutionary significance of social insect symbionts Pp. 340–413 *in* Herman H. R., ed. Social insects. Academic Press, New York, NY, USA.

[ece32276-bib-0027] Kistner, D. H. 1982 The social insects’ beastiary Pp. 2–244 *in* Herman H. R., ed. Social insects. Academic Press, New York, NY, USA.

[ece32276-bib-0028] Krasnov, B. R. , G. I. Shenbrot , I. S. Khokhlova , and A. A. Degen . 2004 Relationship between host diversity and parasite diversity: flea assemblages on small mammals. J. Biogeogr. 31:1857–1866.

[ece32276-bib-0029] Krasnov, B. R. , N. P. Korallo‐Vinarskaya , M. V. Vinarski , G. I. Shenbrot , D. Mouillot , and R. Poulin . 2008 Searching for general patterns in parasite ecology: host identity versus environmental influence on gamasid mite assemblages in small mammals. Parasitology 135:229–242. 17908362 10.1017/S003118200700368X

[ece32276-bib-0030] Lindenfors, P. , C. L. Nunn , K. E. Jones , A. A. Cunningham , W. Sechrest , and J. L. Gittleman . 2007 Parasite species richness in carnivores: effects of host body mass, latitude, geographical range and population density. Glob. Ecol. Biogeogr. 16:496–509.

[ece32276-bib-0031] Lindo, Z. , and N. N. Winchester . 2007 Local‐regional boundary shifts in oribatid mite (Acari: Oribatida) communities: species‐area relationships in arboreal habitat islands of a coastal temperate rain forest, Vancouver Island, Canada. J. Biogeogr. 34:1611–1621.

[ece32276-bib-0032] Lindo, Z. , and N. N. Winchester . 2009 Spatial and environmental factors contributing to patterns in arboreal and terrestrial oribatid mite diversity across spatial scales. Oecologia 160:817–825. 19412624 10.1007/s00442-009-1348-3

[ece32276-bib-0033] Lindo, Z. , N. N. Winchester , and R. K. Didham . 2008 Nested patterns of community assembly in the colonization of artificial canopy habitats by oribatid mites. Oikos 117:1856–1864.

[ece32276-bib-0034] Lussenhop, J. 1976 Soil arthropod response to prairie burning. Ecology 57:88–98.

[ece32276-bib-0035] McArdle, B. H. , and M. J. Anderson . 2001 Fitting multivariate models to community data: a comment on distance‐based redundancy analysis. Ecology 82:290–297.

[ece32276-bib-0036] McLauchlan, K. K. , S. E. Hobbie , and W. M. Post . 2006 Conversion from agriculture to grassland builds soil organic matter on decadal timescales. Ecol. Appl. 16:143–153. 16705968 10.1890/04-1650

[ece32276-bib-0037] Moser, J. C. , and S. R. Blomquist . 2011 Phoretic arthropods of the red imported fire ant in central Louisiana. Ann. Entomol. Soc. Am. 104:886–894.

[ece32276-bib-0038] Oksanen, J. , F. G. Blanchet , R. Kindt , P. Legendre , P. R. Minchin , R. B. O'Hara , et al. 2013 vegan: Community Ecology Package. R package version 2.0‐10. Retrieved from http://cran.r-project.org/package=vegan

[ece32276-bib-0039] Ostfeld, R. S. , C. D. Canham , K. Oggenfuss , R. J. Winchcombe , and F. Keesing . 2006 Climate, deer, rodents, and acorns as determinants of variation in Lyme‐disease risk. PLoS Biol. 4:e145. 16669698 10.1371/journal.pbio.0040145 PMC1457019

[ece32276-bib-0040] Päivinen, J. , P. Ahlroth , V. Kaitala , J. S. Kotiaho , J. Suhonen , and T. Virola . 2003 Species richness and regional distribution of myrmecophilous beetles. Oecologia 134:587–595. 12647132 10.1007/s00442-002-1141-z

[ece32276-bib-0041] Päivinen, J. , P. Ahlroth , V. Kaitala , and J. Suhonen . 2004 Species richness, abundance and distribution of myrmecophilous beetles in nests of *Formica aquilonia* ants. Ann. Zool. Fenn. 41:447–454.

[ece32276-bib-0042] Pérez‐del‐Olmo, A. , M. Fernández , J. A. Raga , A. Kostadinova , and S. Morand . 2009 Not everything is everywhere: the distance decay of similarity in a marine host‐parasite system. J. Biogeogr. 36:200–209.

[ece32276-bib-0043] Phipps, S. J. 2006 Biodiversity of ants (Hymenoptera: Formicidae) in restored grasslands of different ages. Thesis ‐ University of Missouri‐Columbia.

[ece32276-bib-0044] Poulin, R. 2007 Are there general laws in parasite ecology? Parasitology 134:763–776. 17234043 10.1017/S0031182006002150

[ece32276-bib-0045] R Development Core Team . 2015 R: a language and environment for statistical computing. R Foundation for Statistical Computing, Vienna, Austria.

[ece32276-bib-0046] Raffa, K. F. , B. H. Aukema , B. J. Bentz , A. L. Carroll , J. A. Hicke , M. G. Turner , et al. 2008 Cross‐scale drivers of natural disturbances prone to anthropogenic amplification: the dynamics of bark beetle eruptions. Bioscience 58:501–517.

[ece32276-bib-0047] Rettenmeyer, C. W. 1962 Arthropods associated with neotropical army ants with a review of the behavior of these ants (Arthropoda; Formicidae: Dorylinae). Dissertation ‐ University of Kansas.

[ece32276-bib-0048] Rettenmeyer, C. W. , M. E. Rettenmeyer , J. Joseph , and S. M. Berghoff . 2011 The largest animal association centered on one species: the army ant *Eciton burchellii* and its more than 300 associates. Insectes Soc. 58:281–292.

[ece32276-bib-0049] Sanders, D. , and C. Platner . 2007 Intraguild interactions between spiders and ants and top‐down control in a grassland food web. Oecologia 150:611–624. 17091284 10.1007/s00442-006-0538-5

[ece32276-bib-0050] Seastedt, T. R. 1984 Microarthropods of burned and unburned tallgrass prairie. J. Kansas Entomol. Soc. 57:468–476.

[ece32276-bib-0051] Sheldrick, B. H. , and C. Wang . 1993 Particle size distribution Pp. 499–512 *in* Carter M. R., ed. Soil sampling and methods of analysis. Canadian Society of Soil Science, Ottawa, Ontario, Canada.

[ece32276-bib-0052] Uppstrom, K. A. , and H. Klompen . 2011 Mites (Acari) associated with the desert seed harvester ant, *Messor pergandei* (Mayr). Psyche 2011:1–7.

[ece32276-bib-0053] Watling, J. I. , and M. A. Donnelly . 2006 Fragments as islands: a synthesis of faunal responses to habitat patchiness. Conserv. Biol. 20:1016–1025. 16922218 10.1111/j.1523-1739.2006.00482.x

