# Supplementary material for: Species traits and environmental characteristics together regulate ant‐associated biodiversity
Source: Ecol Evol. 2016 Aug 18;6(17):6397–408. doi: 10.1002/ece3.2276 (PMC5016658; doi:10.1002/ece3.2276)
Supplement: Supplementary file 1 — Figure S1. Diagram of sampling methods. Pitfall traps and vegetation quadrats were spaced 25 m apart along the transect. Table S1. Descriptive table of patch‐level variables. Divisions represent general site characteristics, disturbance, and soil variables. Table S2. Abundance and richness of associated mites for ant species collected with baits. Table S3. Mite species collected at the 23 grassland sites. [file ECE3-6-6397-s001.docx]

Supplementary Table 1. Descriptive table of patch-level variables. Divisions represent general site characteristics, disturbance, and soil variables.

| Site | Area (ha) | Traps | Edge:Area | Age  2012 | Time Since Burn 2011 | Time Since Burn 2012 | Litter  Depth  (cm) | Organic Matter  (%) | Bulk Density (g/cm^3^) | Sand  (%) | Clay  (%) | Silt  (%) |
| --- | --- | --- | --- | --- | --- | --- | --- | --- | --- | --- | --- | --- |
| 1 | 1.5 | 5 | 0.04 | 7 | 0 | 1 | 2.53 | 3.6 | 2.8 | 5.7 | 19.4 | 74.9 |
| 2 | 1.6 | 6 | 0.04 | 6 | 5 | 0 | 1.88 | 4.2 | 3.0 | 18.4 | 18.6 | 63.0 |
| 3 | 4 | 7 | 0.03 | 15 | 2 | 3 | 7.28 | 4.5 | 3.2 | 18.6 | 19.8 | 61.6 |
| 4 | 4.9 | 8 | 0.02 | 15 | 1 | 2 | 1.25 | 10.9 | 2.2 | 28.8 | 23.6 | 47.6 |
| 5 | 1.2 | 5 | 0.05 | 5 | 4 | 5 | 4.85 | 4.7 | 3.4 | 13.9 | 26.9 | 59.2 |
| 6 | 17.8 | 10 | 0.01 | 16 | 15 | 16 | 6.48 | 5.8 | 3.0 | 29.6 | 23.6 | 46.8 |
| 7 | 11 | 9 | 0.02 | 5 | 4 | 5 | 1.24 | 8.9 | 2.4 | 12.5 | 34.0 | 53.5 |
| 8 | 5.9 | 8 | 0.02 | 14 | 2 | 0 | 2.04 | 5.7 | 3.0 | 10.2 | 30.2 | 59.6 |
| 9 | 12.1 | 9 | 0.02 | 20 | 3 | 4 | 3.39 | 4.6 | 3.4 | 27.7 | 20.4 | 51.9 |
| 10 | 4.9 | 8 | 0.02 | 5 | 0 | 1 | 1.36 | 4.5 | 3.4 | 46.4 | 19.0 | 34.6 |
| 11 | 12 | 9 | 0.01 | 10 | 3 | 4 | 3.36 | 5.8 | 2.8 | 22.6 | 22.3 | 55.1 |
| 12 | 11.9 | 9 | 0.02 | 24 | 3 | 4 | 6.17 | 5.5 | 3.0 | 14.7 | 22.4 | 62.9 |
| 13 | 12.1 | 9 | 0.01 | 9 | 1 | 2 | 2.00 | 6.5 | 3.0 | 2.1 | 19.4 | 78.5 |
| 14 | 4 | 7 | 0.02 | 6 | 4 | 5 | 5.14 | 4.0 | 3.1 | 14.4 | 21.9 | 63.6 |
| 15 | 1.6 | 6 | 0.04 | 6 | 4 | 5 | 2.67 | 3.6 | 3.6 | 26.5 | 22.8 | 50.7 |
| 16 | 2.8 | 6 | 0.02 | 8 | 7 | 8 | 2.50 | 5.0 | 3.1 | 28.0 | 31.5 | 40.6 |
| 17 | 20.7 | 10 | 0.01 | 11 | 10 | 11 | 3.80 | 3.9 | 2.9 | 7.0 | 20.7 | 72.4 |
| 18 | 16.2 | 10 | 0.02 | 11 | 10 | 11 | 2.29 | 4.7 | 2.6 | 22.1 | 25.3 | 52.7 |
| 19 | 4.5 | 8 | 0.02 | 31 | 1 | 1 | 1.09 | 4.2 | 3.6 | 55.2 | 19.1 | 25.7 |
| 20 | 0.8 | 5 | 0.05 | 11 | 4 | 0 | 1.74 | 5.9 | 3.1 | 47.5 | 16.1 | 36.4 |
| 21 | 2.4 | 6 | 0.02 | 1 | NA | 1 | 1.80 | 3.7 | 2.6 | 6.0 | 17.4 | 76.6 |
| 22 | 8.1 | 9 | 0.02 | 10 | 1 | 2 | 4.53 | 3.7 | 3.6 | 53.1 | 14.4 | 32.5 |
| 23 | 4.9 | 8 | 0.02 | 4 | 3 | 4 | 2.17 | 3.9 | 3.3 | 13.8 | 18.6 | 67.7 |
|  |  |  |  |  |  |  |  |  |  |  |  |  |

Supplementary Figure 1. Diagram of sampling methods. Pitfall traps and vegetation quadrats were spaced 25 m apart along the transect. Paired vegetation quadrats were measured on either side of the pitfall traps adjacent to the transect and averaged. Bait stations were place 8.3 m from each pitfall trap.


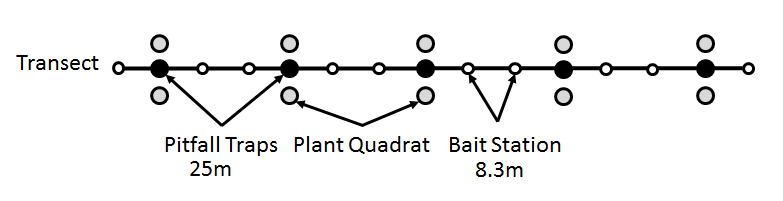


Supplementary Table 2. Abundance and richness of associated mites for ant species collected with baits. The horizontal division separates ant species with and without mites.

| Ant Species | Mite Abundance | Mite Richness | Ant Abundance |
| --- | --- | --- | --- |
| *Monomorium minimum* | 16 | 7 | 82521 |
| *Tapinoma sessile* | 4 | 4 | 5673 |
| *Myrmica americana* | 937 | 18 | 3693 |
| *Lasius neoniger* | 70 | 15 | 2583 |
| *Tetramorium caespitum* | 93 | 5 | 2400 |
| *Aphaenogaster rudis* | 151 | 10 | 1724 |
| *Solenopsis molesta* | 39 | 3 | 1368 |
| *Lasius alienus* | 70 | 10 | 1269 |
| *Myrmica latifrons* | 186 | 8 | 620 |
| *Prenolepis imparis* | 3 | 2 | 336 |
| *Formica pallidefulva* | 11 | 7 | 172 |
| *Formica subsericea* | 2 | 2 | 37 |
| *Formica integra* | 1 | 1 | 19 |
| *Camponotus pennsylvanicus* | 1 | 1 | 7 |
| *Formica rubicunda* | 1 | 1 | 1 |
| *Pheidole tysoni* | 0 | 0 | 1635 |
| *Temnothorax ambiguus* | 0 | 0 | 341 |
| *Nylanderia parvula* | 0 | 0 | 122 |
| *Temnothorax pergandei* | 0 | 0 | 99 |
| *Nylanderia faisonensis* | 0 | 0 | 68 |
| *Forelius pruinosus* | 0 | 0 | 48 |
| *Crematogaster cerasi* | 0 | 0 | 17 |
| *Myrmica pinetorum* | 0 | 0 | 6 |
| *Pheidole pilifera* | 0 | 0 | 4 |
| *Camponotus castaneus* | 0 | 0 | 1 |
| *Camponotus chromaiodes* | 0 | 0 | 1 |
| *Formica postoculata* | 0 | 0 | 1 |
|  |  |  |  |

Supplementary Table 3. Mite species collected at the 23 grassland sites.

| Mite Species | Abundance | Number of Sites | Number of Host Species |
| --- | --- | --- | --- |
| **Astigmata** | 1201 |  |  |
| *Bonomoia* sp1 | 1 | 1 | 1 |
| *Cosmoglyphus* sp1 | 7 | 5 | 1 |
| *Cosmoglyphus* sp2 | 1 | 1 | 1 |
| *Cosmoglyphus* sp3 | 3 | 2 | 2 |
| *Forcellinia* sp1 | 347 | 16 | 8 |
| *Histiostoma* sp1 | 594 | 19 | 7 |
| *Histiostoma* sp2 | 1 | 1 | 1 |
| *Histiostoma* sp3 | 98 | 12 | 1 |
| *Histiostoma* sp4 | 8 | 2 | 1 |
| *Histiostoma* sp5 | 1 | 1 | 1 |
| *Histiostoma* sp6 | 3 | 2 | 1 |
| *Histiostoma* sp8 | 5 | 2 | 1 |
| *Histiostoma* sp9 | 1 | 1 | 1 |
| *Histiostoma* sp10 | 1 | 1 | 1 |
| *Histiostoma* sp11 | 1 | 1 | 1 |
| *Schwiebea* sp1 | 106 | 15 | 8 |
| *Schwiebea* sp2 | 1 | 1 | 1 |
| *Schwiebea* sp3 | 20 | 9 | 7 |
| *Schwiebea* sp4 | 1 | 1 | 1 |
| *Schwiebea* sp6 | 1 | 1 | 1 |
| **Heterostigmata** | 377 |  |  |
| *Bakerdania* sp1 | 1 | 1 | 1 |
| *Imparipes* sp1 | 1 | 1 | 1 |
| *Imparipes* sp2 | 26 | 7 | 2 |
| *Imparipes* sp2 | 33 | 3 | 1 |
| *Imparipes* sp4 | 42 | 4 | 1 |
| *Imparipes* sp5 | 3 | 1 | 2 |
| *Imparipes* sp6 | 2 | 1 | 1 |
| *Petalomium* sp2 | 6 | 1 | 1 |
| *Petalomium* sp3 | 1 | 1 | 1 |
| *Petalomium* sp4 | 12 | 6 | 1 |
| *Scutacarus* sp1 | 41 | 10 | 3 |
| *Scutacarus* sp2 | 29 | 4 | 2 |
| *Scutacarus* sp4 | 150 | 15 | 10 |
| *Scutacarus* sp5 | 2 | 2 | 1 |
| *Scutacarus* sp6 | 1 | 1 | 1 |
| *Scutacarus* sp7 | 1 | 1 | 1 |
| *Scutacarus* sp8 | 1 | 1 | 1 |
| *Scutacarus* sp9 | 2 | 1 | 1 |
| *Scutacarus* sp10 | 1 | 1 | 1 |
| *Scutacarus* sp11 | 11 | 3 | 2 |
| *Scutacarus* sp12 | 4 | 2 | 1 |
| *Scutacarus* sp13 | 1 | 1 | 1 |
| *Tarsonemidae* sp1 | 1 | 1 | 1 |
| *Tarsonemidae* sp2 | 2 | 2 | 1 |
| *Tarsonemidae* sp2 | 1 | 1 | 1 |
| *Tarsonemidae* sp4 | 1 | 1 | 1 |
| *Unguidispus* sp1 | 1 | 1 | 1 |
| **Mesostigmata** | 6 |  |  |
| *Oplitis alienorum* | 1 | 1 | 1 |
| *Oplitis sarcinulus* | 3 | 1 | 1 |
| *Oplitis* nr*. blufftonensis* | 1 | 1 | 1 |
| *Cosmolaelaps* sp1 | 1 | 1 | 1 |
|  |  |  |  |
